# Development of a Tool for Comprehensive Balance Assessment Based on Artificial Intelligence and Anomaly Detection

**DOI:** 10.3390/life15040632

**Published:** 2025-04-10

**Authors:** Márcio Fagundes Goethel, Klaus Magno Becker, Franciele Carvalho Santos Parolini, Ulysses Fernandes Ervilha, João Paulo Vilas-Boas

**Affiliations:** 1 Porto Biomechanics Laboratory, University of Porto, 4200-450 Porto, Portugal; gbiomech@fade.up.pt (M.F.G.); fcsp@ess.ipp.pt (F.C.S.P.); jpvb@fade.up.pt (J.P.V.-B.); 2 Center of Research, Education, Innovation and Intervention in Sport, Faculty of Sport, University of Porto, 4200-450 Porto, Portugal; ulyervil@usp.br; 3 Center for Rehabilitation Research (CIR), School of Health, Polytechnic Institute of Porto, 4200-072 Porto, Portugal; 4 Laboratory of Physical Activity Sciences, School of Arts, Sciences, and Humanities, University of São Paulo, São Paulo 03828-000, Brazil

**Keywords:** balance, anomaly detection, artificial intelligence, artificial neural networks

## Abstract

Falls, a major cause of injury and disability, particularly among older adults, present a significant public-health challenge. Existing methods of balance assessment often lack the sensitivity and specificity needed to identify subtle deviations from normal patterns, hindering early intervention. To address this gap, we introduced a novel artificial intelligence-based tool that leverages anomaly detection to provide a comprehensive assessment of balance performance across all age groups. This study evaluated the tool’s effectiveness in 163 individuals aged 18–85 years who were assessed using a force platform under four conditions: eyes open and eyes closed on firm and foam surfaces. Data analysis, employing an artificial neural network with 19 socio-anthropometric and postural variables, showed the tool’s exceptional accuracy (R = 0.99998) in differentiating among balance profiles. Notably, the model highlighted the significant impact of age and education on balance, with older adults demonstrating increased reliance on visual input, especially when somatosensory information was reduced on foam surfaces. In contrast, younger, more educated individuals exhibited a more integrated sensorimotor approach. These findings demonstrate that our anomaly-detection tool can identify subtle balance impairments often missed by traditional methods, offering valuable insights for personalized fall-risk assessment and intervention. This AI-based approach can provide a holistic assessment of balance, leading to more effective strategies for fall prevention and rehabilitation, particularly in aging populations.

## 1. Introduction

Falls remain one of the most prevalent causes of injury and continue to be a persistent cause of both morbidity and mortality across all age groups [1]. Among elderly people, falls are the second leading cause of accidental death, with those aged 65 and older having higher susceptibility to fatal falls [1]. Often, injuries resulting from falls are associated with reduced physical capacity, loss of independence, and fear of future falls, which in turn can lead to a reduction in physically active living and social engagement [2]. In older populations, the impact of falls on morbidity may be exacerbated by concurrent conditions like osteoporosis, osteopenia, or the use of anticoagulant or antiplatelet medications, which increase the healthcare needs associated with the fall event [3]. As a result of aging, several comorbidities become more frequent, increasing the medical costs associated with such conditions. The functional decline caused by aging leads to motor impairments that are directly related to the increased number of falls, especially in women [4,5,6].

The causes of increased fall risk are well-documented in the literature, involving a combination of muscle weakness, balance impairment, poor gait performance, vision and hearing loss, and the use of psychotropic medications [7]. The natural aging process leads to loss of balance due to the loss of the muscle capacity to resist external forces and thus maintain balance, with consequent instability in terms of dynamic stability, static stability, anticipatory postural control, sensory integration, functional stability limits, reactive postural control, and cognitive factors [5,8].

This complex interplay of factors has led to a vast array of standardized postural-control assessments. Over the years, various measures have attempted to address the limitations of previous balance assessments by analyzing center of pressure (COP) deviation and area [9], COP velocity within the time domain [10], entropy within the complexity domain [11,12], and mean and median frequency within the frequency domain [13,14]. The major issue is that due to this wide range of assessments, which address specific problems, the variables offer only partial information, omitting integrated data about balance and yielding simple global measures that are insufficient to provide the insights necessary to predict the environments and particular situations that may result in failure of postural control [15]. These approaches may not be able to differentiate among various underlying causes of balance impairments and therefore may not be suitable for predicting falls in real-world settings, considering that no variable occurs alone in real situations. It is recommended, therefore, to conduct assessments that can effectively and comprehensively identify deficiencies in postural control so that strategies and exercise programs that are effective in fall prevention can be proposed [16].

Machine learning and deep learning algorithms have proven highly effective in analyzing complex, high-dimensional data, particularly in scenarios where traditional statistical approaches may fail to capture intricate patterns [17]. These methods can identify nonlinear relationships and adaptively learn from data without requiring prior assumptions, making them suitable for diverse populations and conditions [18]. To achieve a deeper understanding of deficiencies in postural control, researchers have turned to mathematical modelling and artificial intelligence as powerful tools [13,15,17]. Recent studies have demonstrated the growing utility of machine learning in the medical field. For instance, one study discussed the use of deep learning algorithms for the detection of subtle balance impairments in older adults, highlighting their potential to improve fall-risk prediction and personalized interventions [19]. Another research effort explored the application of machine learning techniques to the analysis of gait and postural control, emphasizing their ability to capture complex, nonlinear interactions among various factors influencing balance [20]. These findings underscore the promise of artificial intelligence for advancing our understanding of balance control and fall-prevention strategies. Specifically, anomaly-detection algorithms, such as the one used in this study, are well-suited for identifying subtle changes in motor patterns that may indicate balance impairments [21]. The ability of these techniques to process and analyze large volumes of data allows for a more comprehensive understanding of balance performance across various age groups and conditions [17,21].

Machine learning and deep learning algorithms offer the ability to analyze small and large amounts of data and uncover hidden patterns and associations that may not be evident through traditional statistical approaches [22]. One of the main advantages of using artificial intelligence to study the relationship between motor control and balance is its ability to capture and analyze multiple dimensions of balance. We used an anomaly-detection algorithm, as they can detect unusual or unforeseen patterns in data that may result in superior performance in terms of loss values and mean squared error; it thus may be able to detect subtle changes in motor patterns. Since the algorithm can learn from data without prior knowledge, it is adaptable to different populations and conditions. Unlike previous studies that focused primarily on specific age groups or predefined balance deficits, this study used a novel, age-agnostic approach employing anomaly detection to identify deviations from the ‘normal’ balance pattern, providing a broader assessment of balance performance.

Therefore, the aim of the present study was to achieve a comprehensive assessment of balance performance, regardless of the participant’s age. To this end, we proposed the development of an artificial intelligence-based tool capable of identifying and quantifying deviations from the normal balance pattern in both young and elderly individuals.

## 2. Materials and Methods

The data were extracted from a public database [23]. Data collection for each participant took place in a single session lasting one to two hours. Each participant was assessed by the same experienced examiner (D.A.S.) at the Biomechanics and Motor Control Laboratory of the Federal University of ABC, Brazil. Prior to the evaluations composing this dataset, pilot studies were conducted with five participants for equipment training and familiarization with the experimental protocol. Data from these participants are not included in this dataset. The study was approved by the local ethics committee of the Federal University of ABC (#842529/2014).

### 2.1. Sample

The sample included 163 participants (116 women and 47 men) [23]. Out of the 163 participants, 16 were classified as having at least one severe disability (eight with auditory and vestibular deficits, two with visual deficits, three with musculoskeletal deficits, and one with visual and musculoskeletal deficits). Ages ranged from 18 to 85 years, with body masses ranging from 44.0 to 75.9 kg, heights from 140.0 to 189.8 cm, and body mass indices (BMI) from 17.2 to 31.9 kg/m^2^.

### 2.2. Data Collection

Data from the balance trials were collected using a commercial force platform measuring 40 cm × 60 cm (OPT400600-1000; AMTI, Watertown, MA, USA) and an amplifier (Optima Signal Conditioner; AMTI, Watertown, MA, USA) with a sampling frequency of 100 Hz. The force platform was factory-calibrated and exhibited an average CoP accuracy of 0.02 cm. The fluctuation in COP displacement (indicative of measurement precision) was estimated as the standard deviation value of COP data when a static load of 30 kg was placed on the force platform for 30 s; the resulting value was 0.005 cm.

Data acquisition was conducted using NetForce software (Version 3.5.3; AMTI, Watertown, MA, USA). NetForce software generates calibrated forces and moments of force (Fx, Fy, Fz, Mx, My, Mz) from the platform into a proprietary binary file format. All subsequent steps, including reading these binary files, data processing, analysis and visualization, and data export to text files, were implemented in the Python (1.15.2 Version) language using the SciPy Stack [22,24]. The force-platform data were smoothed using a fourth-order zero-lag Butterworth low-pass filter with a cutoff frequency of 10 Hz, and the centers of pressure in the anterior–posterior (COPx—positive is anterior) and medial–lateral (COPy—positive is right) directions were calculated according to standard formulas [23]. It is important to note that the force-platform data are expressed as forces and moments of force in the coordinate system of the force platform and refer to the forces and moments of force that the participant is applying to the force platform. This contrasts with ground reaction forces, where the forces act on the participant [25].

### 2.3. Procedures

The stabilometric assessment was based on the most common practices utilized in research laboratories and clinical settings [22,23]. Participants’ balance was assessed while they stood for 60 s under each of four different conditions: on a rigid surface with eyes open; on a rigid surface with eyes closed; on an unstable surface (a foam block with a height of 6 cm (Balance Pad; Airex AG, Sins, Switzerland)) with eyes open; and on an unstable surface with eyes closed. Each condition was tested three times, and the order of the conditions was randomized among participants. Randomization was conducted by the examiner before data collection using a computerized random-number generator.

In all conditions, participants were instructed to stand barefoot and as still as possible, with their arms by their sides, and to fix their gaze on a round black target measuring 5 cm in diameter, placed at eye level on a wall 3 m away. For trials in which participants kept their eyes closed, they were instructed to initially look at the target and keep their eyes open, establish a stable and comfortable posture given the requirements, and then close their eyes a few seconds later, initiating data acquisition.

### 2.4. Anomaly-Detection Structure

**Figure 1:**
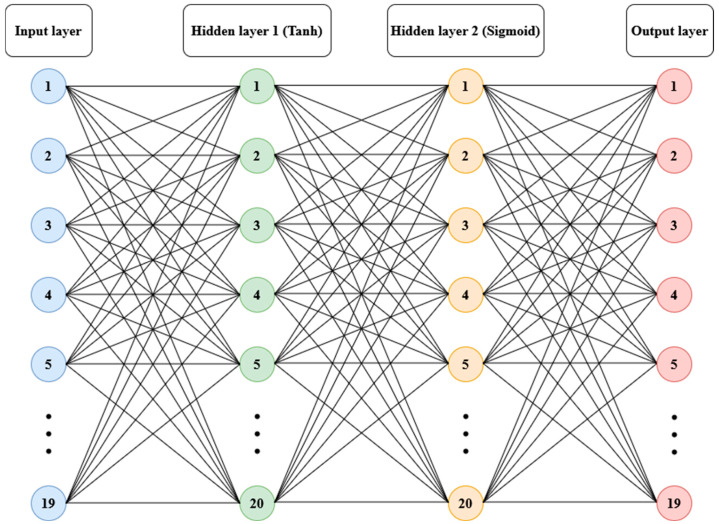
Structure of artificial neural network model: 19 variables in the input layer; hyperbolic tangent (Tanh) sigmoid corresponding to two hidden layers with 20 neurons each and the output layer.

Anomaly detection refers to the problem of finding patterns in data that do not conform to expected behavior [21]. To model the relationships among the variables that contribute to balance, data from 156 participants (112 women and 44 men, ages between 18.8 and 85.8 years, 162.5 ± 9.68 cm in height, 62.19 ± 8.14 kg in body mass) from the referenced public dataset were used [23]. An artificial neural network structure with two hidden layers was trained, utilizing hyperbolic tangent and sigmoid logistic transfer functions, respectively, with 20 neurons in each layer and full connectivity [26]. The input layer consisted of 19 variables covering socio-anthropometric dimensions (age, gender, body mass, height, body mass index, foot length, years of study) and balance performance in bipedal force-platform tests with durations of 60 s, conducted with eyes open and eyes closed on a firm surface and on a foam surface (CoP area, mean velocity, and median frequency). The output layer contained the same information but with the order of participants randomized. The learning algorithm used was Bayesian regularization backpropagation [26] (see Figure 1).

After 494 epochs through an established checkpoint (total of 10,000 epochs), the best performance value (mean squared error = 0.0711) was reached. The training performance resulted in a coefficient of determination (R^2^) of 0.99998; testing performance yielded an R^2^ of 0.93509, and the R^2^ for all samples was 0.98697. The data of all the participants were then simulated using the model obtained and the estimates were compared with the real data through a single linear regression. The R^2^ generated was then normalized through its subtraction from the minimum value found and subsequently divided by the value of the difference between the maximum value and the minimum value. Three subgroups were created based on their R^2^ normalized position in relation to the 25th and 75th percentiles. The first group consisted of participants with values below the 25th percentile; the second group consisted of participants with values between the 25th and 75th percentiles; and the third group was composed of participants with values above the 75th percentile. The groups were then compared using analysis of variance (ANOVA), with subsequent pairwise comparisons between groups adjusted using the Bonferroni correction. Assumptions of normality and homogeneity of variances was tested using the Shapiro–Wilk test and Levene’s test, respectively. An alpha value of 0.05 was used for all statistical tests.

## 3. Results

**Figure 2:**
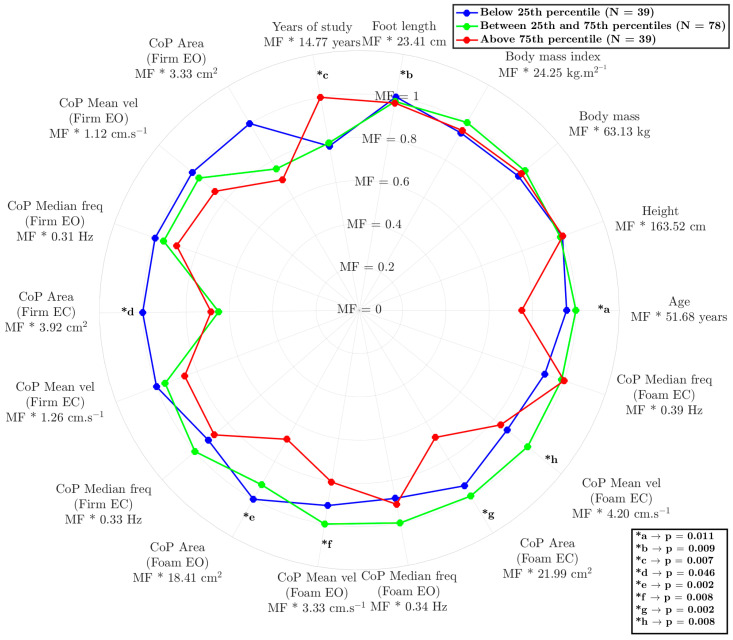
Radar chart comparing postural control and anthropometric characteristics among three groups: below the 25th percentile (green), between the 25th and 75th percentiles (blue), and above the 75th percentile (red). The layers of radar correspond to the multiplication factor (MF) of the variable’s value. The asterisks (*) indicate statistically significant differences between groups in the variance analysis, with the corresponding *p*-values provided in the legend. “MF” represents the mean ± standard deviation for each variable. CoP = center of pressure; EO = eyes open; EC = eyes closed.

The model reflected interesting differences between the groups (Figure 2), demonstrating its ability to understand the relationship between age, anthropometry, and sensorimotor information. The results are expressed as the multiplication factor (MF) of each condition multiplied by the constant value (as mean) of each variable.

The influences of age (*p* = 0.025 and *p* = 0.010), years of education (*p* = 0.011 and *p* = 0.021), and the reduced area of the CoP on the foam surface under eyes-open conditions (*p* = 0.003 and *p* = 0.011) and eyes-closed conditions (*p* < =0.001 and *p* = 0.033) were examined. The results highlight a significant differentiation between the participants in the highest-percentile position and other participants (25th and 50th compared to 75th), indicating that these variables play a crucial role in human balance, triggering sensory responses beyond the visual component. Notably, visual deprivation had a unique discriminatory capacity; it was associated with values below the 25th percentile, with statistically better results only for CoP area measured on the firm surface with eyes closed (*p* = 0.002 and *p* = 0.048).

## 4. Discussion

Human balance is a dynamic process, a continuous interplay between the muscular, neurological, and sensory systems. Each one of these systems experiences age-related decline at different rates, contributing to a complex and multifaceted degradation of balance over time. In recognition of this complexity, our research method did not rely on traditional, single-domain, or individualized-comparison approaches to balance assessment. Instead, we applied an artificial intelligence model capable of integrating a wide array of balance-related variables drawn from diverse populations and encompassing multiple physiological domains. This model employed anomaly detection to establish a comprehensive, individualized profile of balance performance. By learning the unique ‘signature’ of an individual’s balance, our approach appears to surpass the limitations of conventional methods, which often lack the sensitivity and specificity required to capture the nuances of this multidimensional process.

This AI-driven approach represents a paradigm shift in balance assessment, moving beyond incremental advancements to a fundamentally new way of understanding this complex process. As illustrated in Figure 2, our anomaly-detection approach demonstrates a remarkable ability to decipher the intricate relationships among various factors influencing balance [27]. The study results appear to provide evidence for the significant impacts of age, education level, and CoP area on balance performance, particularly under conditions of altered visual input (eyes open/closed) and surface stability (hard/soft). By considering the contributions of these sensory systems to balance perception and maintenance, we can begin to unravel how factors like age, anthropometry, and education level shape an individual’s unique balance profile. For instance, while Winter [28] conventionally described CoP as a neuromuscular response to imbalances in the body’s center of mass, our findings reveal a more nuanced picture. Notably, as shown in Figure 2, the AI tool effectively identified individuals with scores below the 25th percentile based on subtle CoP variations detected when they were standing on rigid surfaces with eyes open, even though these differences did not reach statistical significance using traditional methods. This highlights the tool’s enhanced sensitivity in detecting subtle balance impairments that might otherwise be missed, suggesting potential for earlier and more personalized interventions.

This potential is particularly relevant when considering the well-established impact of aging on balance control. Aging is associated with numerous changes in the neuromuscular and neurosensory systems, which lead to increased postural sway, a factor often reflected in a larger area of CoP displacement [5]. Our findings align with this understanding, as illustrated in Figure 2, where participants above the 75th percentile, who also had the lowest average age, exhibited superior balance performance. Interestingly, in addition to age, education level emerged as a significant factor. Participants above the 75th percentile also demonstrated a higher level of education. Indeed, education and engagement in challenging cognitive activities have been associated with greater brain plasticity, particularly in areas of the brain involved in motor control and sensory integration [29,30,31]. This result supports the idea of cognitive reserve, where a more robust cognitive machinery may offer a protective effect against age-related balance declines. This enhanced plasticity may allow for more efficient adjustments and adaptations in response to changing environmental conditions or internal perturbations, ultimately contributing to improved balance performance. This interplay between age, education, and balance underscores the complex nature of postural control and highlights the need for multifaceted assessment approaches, such as that provided by our machine learning-based approach.

However, the relative contributions of different sensory systems to balance control, particularly in the context of aging and cognitive factors, remain an active area of investigation. It is well established that vision plays a dominant role in maintaining postural stability [32,33], although the precise nature of the crossmodal interactions involving dynamic stimuli is not fully understood [34]. Postural control fundamentally relies on the integration of information from the vestibular, visual, and somatosensory systems [30,32], and in individuals with balance disorders, the reliance on visual input may be even more pronounced [32]. Our data support this paradigm, as illustrated in Figure 2: when participants’ eyes were closed, the AI model effectively differentiated the lowest 25th percentile (representing poorer balance) from the other groups based on CoP area (*d). This suggests that individuals with poorer balance may rely more heavily on visual information to compensate for deficits in other sensory systems. This increased reliance on vision may be linked to age-related declines in the concentration of sensory receptors, the number of synapses, axons, and dendrites, and reduced nerve-conduction velocity, all of which can contribute to balance impairment in older adults [30].

Beyond visual dependence, our findings also highlight the crucial role of somatosensory input in maintaining balance, particularly when visual information is limited or unavailable. The anomaly model revealed that surface differences (hard vs. soft) were responsible for differentiating the two highest-performing balance groups (25th–75th percentile and above 75th percentile), as indicated by CoP area measured with both eyes open and both eyes closed (*e and *g). These results indicate that younger, more educated adults may more effectively integrate somatosensory information for balance, even when visual input is compromised [30]. This ability to integrate sensory information effectively may be linked to the concept of cognitive reserve, where a higher level of education and cognitive engagement contributes to greater brain plasticity [29,35]. As individuals age, various cognitive functions, including attention, processing speed, and short-term memory, may decline [36]. These age-related changes can, in turn, impact the ability to effectively integrate sensory information and generate appropriate motor responses to maintain balance, particularly in challenging situations such as navigating uneven surfaces or performing tasks in low-light conditions.

Our findings offer some intriguing insights into the potential interplay between aging, sensory function, and balance performance.

Our results suggest that the somatosensory system is the first to be affected by aging, as evidenced by the 75th-percentile group exhibiting smaller CoP displacement on the foam surface, even with eyes closed. This suggests that aging may diminish the ability to effectively use somatosensory information for balance, leading to increased reliance on visual cues. Notably, this deterioration in sensory function can be mitigated by education [35], highlighting a potential strategy to counterbalance the effects of aging. Additionally, our study’s result underscores the power of an AI-driven approach to balance assessment. The model was able to identify complex, nonlinear relationships among a multitude of factors affecting balance, allowing for a more nuanced, individualized understanding of balance performance. These findings align with the growing focus on personalized healthcare, suggesting that interventions tailored to an individual’s unique balance profile, as determined by our AI, could be more effective than conventional approaches.

**Study Limitations**

While the AI-based approach offers significant advancements in balance assessment, several methodological limitations must be acknowledged. The external validity of the model is limited due to the sample’s potential lack of representativeness in terms of health status. Additionally, the model has not been validated with independent cohorts, restricting its generalizability and clinical applicability. Therefore, further testing is needed to confirm the model’s accuracy. Furthermore, psychological factors, such as fear of falling and balance confidence, were not considered, and these elements may influence postural control. These limitations emphasize the necessity for further research to validate the model and refine its application in diverse settings.

**Future research**

To strengthen the clinical and practical relevance of this AI model, future studies should prioritize external validation in larger, more diverse populations. It is also crucial to assess the model’s reliability across various contexts, particularly for individuals with balance disorders or older adults at high risk of falls. Integrating cognitive and psychological factors into the model could enhance its comprehensiveness in evaluating balance control. Additionally, research should explore the potential of personalized interventions based on AI-generated balance profiles, assessing whether early detection of balance impairments leads to more effective prevention and rehabilitation strategies. Finally, conducting direct comparisons between this model and traditional assessment methods would clarify its clinical value and support a data-driven, personalized approach to balance management.

## 5. Conclusions

The development of a comprehensive balance-assessment tool that uses artificial intelligence based on anomaly detection represents a significant advancement in the field of biomechanics and healthcare in that it unveils the intricate relationships among various factors that appear to influence balance. Notably, our findings suggest a heightened reliance on visual input in individuals with poorer balance, possibly as a compensatory mechanism for age-related decline in other sensory systems. This insight, coupled with the observed link between education and balance performance, highlights the potential role of cognitive reserve in mitigating age-related balance impairments. These findings can enhance our understanding of balance mechanisms and facilitate personalized interventions and fall prevention by considering the sensory and neural factors related to balance.

## Data Availability

All the data is available at Figshare (DOI: 10.6084/m9.figshare.4525082) from a public dataset [23].
